# Knowledge, attitudes, and practices of adolescent depression among parents of children diagnosed with depression in Ningbo City, eastern China

**DOI:** 10.3389/fpubh.2024.1404819

**Published:** 2024-06-11

**Authors:** He Gao, Ni Dai, Chen Lin, Yun Ye, Dalu Yang, Qingyu Zhang, Jiaxin Mao, Zhongze Lou, Yunxin Ji, Liemin Ruan, Yanbin Hou

**Affiliations:** ^1^Department of Psychiatry, The Affiliated Kangning Hospital of Ningbo University, Ningbo, China; ^2^Department of Psychosomatics, The First Affiliated Hospital of Ningbo University, Ningbo, China

**Keywords:** knowledge, attitudes, practices, adolescent depression, parent

## Abstract

**Objective:**

To investigate parental knowledge, attitudes, and practices (KAP) toward adolescent depression.

**Methods:**

A cross-sectional survey was conducted between October 2022 and October 2023 at The First Affiliated Hospital of Ningbo University among parents of adolescents diagnosed with depression. A self-administered questionnaire was used to collect the parents’ demographic characteristics and KAP toward adolescent depression.

**Results:**

A total of 522 questionnaires were collected from parents of depressed adolescents. Among the participants, 383 (73.37%) were female. In addition, 426 participants (81.61%) had children aged 14–18. The mean knowledge, attitude, and practice scores were 9.09 ± 2.37 (possible range: 0–12), 37.04 ± 4.11 (possible range: 11–55), and 31.53 ± 3.84 (possible range: 8–40), respectively. There were significant positive correlations between knowledge and attitude (*r* = 0.225, *p* < 0.001), knowledge and practice (*r* = 0.240, *p* < 0.001), and attitude and practice (*r* = 0.381, *p* < 0.001). The path analysis showed significant direct effects of knowledge on attitude (β = 0.422, *p* < 0.001) and practice (β = 0.283, *p* < 0.001). There was an indirect effect of knowledge on practice through attitude (β = 0.131, *p* = 0.004). Attitude directly impacted practice (β = 0.311, *p* < 0.001).

**Conclusion:**

Parents of adolescents diagnosed with depression exhibited moderate KAP regarding adolescent depression. The study underscored the importance of targeted interventions to improve parental KAP in supporting adolescents with depression. Moreover, future research should explore additional factors influencing parental attitudes and behaviors toward adolescent depression to develop more effective interventions.

## Introduction

Globally, as of January 2020, the World Health Organization (WHO) reported that more than 264 million individuals across various age groups were grappling with depression. Of particular concern is adolescent depression, affecting one in five individuals by the age of 18 (1, 2). This pervasive issue poses significant threats to academic performance and social relationships and increases the risks of substance abuse and suicide (3). Effective parent-adolescent communication is a protective factor against adolescent depression, while contentious relationships between parents and adolescents amplify the likelihood of depression in adolescents (4). The correlation between heightened conflict levels and adolescent depression is evident, emphasizing the critical role of parental responses in shaping emotional functioning and management. The gravity of adolescent depression extends beyond immediate consequences, casting a shadow over both current and future mental well-being, underscoring the urgency of addressing this issue. It is particularly crucial considering the escalating prominence of adolescent depression as a public health concern in China (4, 5).

The knowledge, attitude, and practice (KAP) theory is a fundamental framework for shaping health behaviors among individuals (6). This theoretical model is a comprehensive tool for assessing the KAP of a target population toward a healthcare domain (7). In addition, evaluating the KAP using a questionnaire facilitates the evaluation of the demand for and acceptance levels of pertinent health-related content. Embedded within health literacy, the KAP model is based on the core tenet that knowledge positively influences attitudes, shaping individual practices (8). The adolescent period is a critical juncture for the onset of depression, with many individuals experiencing depressive symptoms for the first time during this developmental stage (5). Understanding parental cognition and responses to depression during adolescence proves instrumental in the early detection and intervention of potential depressive issues. Given the profound impact of the family environment and parental roles on adolescent mental health, some parents may hold misconceptions about adolescent depression, potentially causing their children to feel shame or have suboptimal support. Adolescents are at a crucial stage of psychological and physiological development and are highly susceptible to the influence of family dynamics. Negative parental attitudes and behaviors can significantly impact the management of adolescent depression, potentially impeding recovery.

Previous KAP studies on depression focused on various populations, including healthcare providers and the general public (9–11), and there is a noticeable research gap specific to adolescent depression. Therefore, this study aimed to investigate the KAP toward adolescent depression among parents of children diagnosed with depression.

## Methods

### Study design and participants

This cross-sectional study was conducted between October 2022 and October 2023 at The First Affiliated Hospital of Ningbo University Hospital among parents of adolescents diagnosed with depression.

The inclusion criteria for parents who completed the questionnaires were (1) participants whose adolescent child had received a diagnosis of major depressive disorder based on [DSM-5] [DSM-5] (12), (2) participants who volunteered participation, and (3) participants who provided written informed consent. The exclusion criteria were (1) participants with limited reading/writing proficiency and/or (2) participants who themselves were affected by mental health disorders.

The study was approved by the Ningbo First Hospital Ethics Committee (Ethical Approval Number: 2022-016A). Written informed consent was obtained from all participants.

### Questionnaire

The questionnaire was designed based on a previous study (13) and reviewed by two highly experienced chief physicians, each with >20 years of professional experience in psychiatry. A pilot study was conducted (62 complete questionnaires) to assess the reliability of the questionnaire. The result showed a Cronbach’s α coefficient = 0.712, indicating an acceptable internal consistency. Throughout the assessment process led by the experienced chief physicians, the questionnaire underwent thorough scrutiny to ensure correctness and appropriateness. Any items deemed incorrect or inappropriate were removed to enhance content validity. Furthermore, during the pilot study, the participants were encouraged to raise questions about any items they found confusing or unclear, with researchers providing clarifications as necessary. Subsequently, no items were perceived as confusing after explanation, suggesting face validity.

The final questionnaire was in Chinese and encompassed four dimensions, totaling 45 items. These dimensions include demographic characteristics (13 items), knowledge (12 items), attitudes (11 items), and practices (9 items). The knowledge dimension used a binary scoring system (1 for correct, 0 for incorrect or unclear responses). The correctness rates were determined for each knowledge item. The attitude and practice dimensions used a 5-point Likert scale. The knowledge dimension scores ranged from 0 to 12, the attitude dimension from 11 to 55 (with reverse-scoring for questions A1, A2, A3, A4, A9, A10, and A12), and the practice dimension from 8 to 40 (with reverse-scoring for questions P6 and P7; question P9 was descriptive only).

The questionnaire collection included paper-based questionnaires and digital dissemination via QR codes accessible through the WeChat App. One parent of an adolescent with depression could complete the questionnaire. This approach aimed at reaching a convenience sample of parents. In order to ensure informed participation, the potential participants were provided with an overview of the study’s objectives before completing the questionnaire, and it was emphasized that their responses would remain anonymous.

Several measures were implemented to maintain the integrity and quality of the data collected. Firstly, the number of responses from the same WeChat ID was restricted for each question to avoid duplicate entries. A mandatory response design was utilized to eliminate the occurrence of missing values. Two investigators meticulously reviewed all responses to enhance data quality. As part of the quality control process, questionnaires that contained logical inconsistencies (e.g., impossible age) or displayed a pattern of selecting the same option for all questions were identified and excluded from the analysis.

Bloom’s classification was adopted to evaluate the participants’ KAP levels. Specifically, participants scoring above 80% were categorized as having adequate knowledge, positive attitudes, and proactive practices. Those within the 60 to 80% range were classified as having moderate levels. Scores below 60% indicated inadequate knowledge, negative attitudes, and inactive practices (14).

### Statistical analysis

SPSS 26.0 was used for statistical analysis (IBM, Armonk, NY, United States). The continuous data were presented as means ± standard deviations (SD); those confirmed to meet the normal distribution (according to the Kolmogorov–Smirnov test) were compared using Student’s *t*-test or ANOVA, while those with a skewed distribution were compared using the Wilcoxon Mann–Whitney test or the Kruskal-Wallis test. The categorical data were expressed as *n* (%). Spearman’s correlation analysis was conducted to assess the correlations among KAP dimensions. A pathway analysis was performed to explore the direct and indirect relationships among the three dimensions. A post-hoc analysis for construct validity was conducted using the Kaiser-Meyer-Olkin (KMO) analysis and confirmatory factor analysis (CFA). A two-sided *p* < 0.05 was considered statistically significant.

## Results

In this study, 522 questionnaires were collected from parents of adolescents diagnosed with depression. The questionnaire demonstrated good construct validity (KMO = 0.817, *p* < 0.001). The CFA indicated that the questionnaire had a good fit (Supplementary Figure S1 and Supplementary Table S1). Among the participants, 383 (73.37%) were female, 388 (74.33%) were > 40 years of age, 184 (35.25%) had junior high school education, and 268 (51.34%) had only one child. Concerning the adolescents with depression, 368 (70.50%) of the participants’ children were girls. In addition, 426 (81.61%) of the children experienced depression between the ages of 14 and 18, with a mean diagnosis history of 13.66 ± 16.79 months. Moreover, 85 (35.44%) children exhibited moderate depression. The mean KAP scores were 9.09 ± 2.37 (possible range: 0–12), 37.04 ± 4.11 (possible range: 11–55), and 31.53 ± 3.84 (possible range: 8–40), respectively. The KAP scores varied among parents based on residence (*p* < 0.001, *p* = 0.013, and *p* < 0.001) and education (*p* < 0.001, *p* = 0.033, and *p* < 0.001). In addition, gender (*p* = 0.001), occupation (*p* = 0.001), monthly *per capita* income (*p* = 0.001), number of children (*p* < 0.001), and the severity of the child’s depression (*p* = 0.012) were associated with variations in the knowledge scores. Gender differences appeared to impact attitude (*p* = 0.004), while differences in the number of children influenced practice (*p* = 0.006) (Table 1).

**Table 1:** Demographic characteristics and KAP.

	*N* (%)	Knowledge, mean ± SD	*p*-value	Attitude, mean ± SD	*p*-value	Practice，mean ± SD	*p*-value
**Total score**		9.09 ± 2.37		37.04 ± 4.11		31.53 ± 3.84	
**Age, years**			0.362		0.238		0.998
18–40	134 (25.67)	8.93 ± 2.48		36.68 ± 3.73		31.53 ± 3.64	
>40	388 (74.33)	9.15 ± 2.33		37.16 ± 4.23		31.53 ± 3.91	
**Gender**			0.001		0.004		0.238
Male	139 (26.63)	8.53 ± 2.52		36.19 ± 4.37		31.20 ± 3.88	
Female	383 (73.37)	9.30 ± 2.28		37.35 ± 3.97		31.65 ± 3.82	
**Residence**			<0.001		0.013		<0.001
Rural	152 (29.12)	8.38 ± 2.59		36.23 ± 4.25		30.55 ± 4.16	
Urban	284 (54.41)	9.52 ± 2.12		37.31 ± 3.98		32.08 ± 3.66	
Suburban	86 (16.48)	8.95 ± 2.44		37.58 ± 4.11		31.47 ± 3.53	
**Education**			<0.001		0.033		<0.001
Primary school or below	42 (8.05)	7.79 ± 2.74		35.19 ± 4.68		28.95 ± 4.28	
Junior high school	184 (35.25)	8.46 ± 2.37		36.98 ± 3.99		31.16 ± 3.74	
High school/Technical school	122 (23.37)	9.07 ± 2.43		37.33 ± 4.17		32.08 ± 3.81	
College/Bachelor’s	157 (30.08)	10.12 ± 1.79		37.41 ± 3.98		32.24 ± 3.70	
Master’s and above	17 (3.26)	9.82 ± 1.81		36.82 ± 3.71		31.47 ± 1.97	
**Occupation**			0.001		0.351		0.824
Government officials, leaders of state-owned enterprises and institutions	17 (3.26)	9.65 ± 2.23		36.65 ± 3.59		32.06 ± 3.88	
Professionals (teachers, doctors, engineers, writers, etc.)	63 (12.07)	9.63 ± 2.17		36.71 ± 3.60		31.63 ± 3.88	
General clerical and related personnel	133 (25.48)	9.41 ± 2.47		37.24 ± 4.13		31.56 ± 3.92	
Business and service personnel	97 (18.58)	8.73 ± 2.26		37.19 ± 3.50		31.93 ± 3.98	
Agricultural, forestry, animal husbandry, fishery, and water conservancy production personnel	13 (2.49)	7.54 ± 1.85		35.08 ± 4.35		31.85 ± 4.90	
Production, transportation equipment operators, and related personnel	33 (6.32)	8.00 ± 2.41		36.03 ± 3.79		31.03 ± 4.24	
Others	166 (31.80)	9.13 ± 2.34		37.31 ± 4.64		31.26 ± 3.53	
**Monthly income, Yuan**			0.001		0.289		0.118
<2000	24 (4.60)	8.75 ± 2.72		36.67 ± 5.10		30.25 ± 4.01	
2000–5,000	148 (28.35)	8.42 ± 2.57		36.53 ± 4.15		31.05 ± 3.88	
5,000–10,000	196 (37.55)	9.33 ± 2.15		37.45 ± 3.83		31.74 ± 3.87	
10,000–20,000	87 (16.67)	9.41 ± 2.26		36.86 ± 4.20		31.79 ± 3.58	
>20,000	67 (12.83)	9.61 ± 2.24		37.33 ± 4.26		32.09 ± 3.83	
**Marital status**			0.609		0.531		0.550
Married	460 (88.12)	9.12 ± 2.33		37.08 ± 4.13		31.53 ± 3.82	
Divorced	52 (9.96)	9.00 ± 2.68		37.00 ± 4.06		31.75 ± 4.19	
Widowed	10 (1.92)	8.40 ± 2.37		35.60 ± 3.50		30.30 ± 2.50	
**Number of children**			<0.001		0.258		0.006
1	268 (51.34)	9.56 ± 2.22		37.08 ± 4.31		31.91 ± 3.64	
2	223 (42.72)	8.69 ± 2.40		37.16 ± 3.84		31.33 ± 3.95	
3 or 4	31 (5.94)	7.97 ± 2.52		35.87 ± 4.11		29.71 ± 4.23	
**Age of child who suffers from depression**			0.056		0.051		0.363
<14 years	96 (18.39)	8.68 ± 2.78		36.30 ± 4.36		31.21 ± 4.12	
14–18 years	426 (81.61)	9.19 ± 2.26		37.21 ± 4.03		31.60 ± 3.77	
**Gender of child who suffers from depression**			0.953		0.160		0.640
Male	154 (29.50)	9.08 ± 2.19		36.65 ± 4.29		31.41 ± 4.08	
Female	368 (70.50)	9.10 ± 2.44		37.20 ± 4.02		31.58 ± 3.74	
**Duration of child’s depression**	13.66 ± 16.79						
**Severity of child’s depression**			0.012		0.069		0.201
Mild	154 (29.50)	9.29 ± 2.37		36.75 ± 4.13		31.63 ± 3.70	
Moderate	185 (35.44)	9.38 ± 2.30		36.95 ± 4.05		31.90 ± 3.88	
Severe	87 (16.67)	8.69 ± 2.53		36.67 ± 4.50		30.92 ± 4.02	
Unclear	96 (18.39)	8.58 ± 2.25		38.02 ± 3.69		31.22 ± 3.77	
**Medical insurance type for child who suffers from depression**			0.728		0.840		0.514
One type	490 (93.87)	9.10 ± 2.33		37.07 ± 4.13		31.49 ± 3.84	
Two types	9 (1.72)	9.56 ± 2.40		36.67 ± 3.84		31.44 ± 4.22	
Uninsured	23 (4.41)	8.83 ± 3.13		36.61 ± 3.73		32.43 ± 3.85	

The distribution of the knowledge scores revealed that the three knowledge items with the highest correctness rates were as follows: “*Early diagnosis and timely treatment of depression can improve treatment outcomes.*” (K7) with 95.02%, “*Signs like suicidal statements, suicide attempts, worsening depression or anxiety, restlessness, panic attacks, insomnia, irritability, impulsiveness, and manic behavior suggest a worsening of depression and the need for prompt medical attention.*” (K6) with 94.06%, and “*Behaviors such as maintaining a regular diet, engaging in physical activity, getting sufficient sleep, developing interests, and communicating with others can have a positive impact on improving depression.*” (K12) with 93.87%. The three items with the lowest correctness rates were “*Antidepressant medications are addictive.*” (K10) with 28.93%, “*Having many friends does not necessarily protect a child from developing depression.*” (K4) with 52.49%, and “*It is essential to follow a medical professional’s guidance when discontinuing antidepressant medication, even if one’s mood improves.*” (K11) with 52.49% (Supplementary Table S2).

The parents who participated in the study had different attitudes toward their child’s illness. Indeed, 67.24% expressed that they were very worried (A1). However, 47.7% felt it bothered them (A2). Alternatively, 71.46% reported that it did not make them ashamed (A3). 53.26% were very keen to understand why their child was depressed (A5), and 58.43% strongly agreed with the importance of respecting their child and encouraging them to express their emotions (A7). Regarding the side effects of antidepressant medication, such as the impact on growth, 56.9% reported varying degrees of concern (A10). However, it was encouraging to note that 95.98% of the respondents expressed trust in the advice provided by the physicians (A11) (Supplementary Table S3).

In terms of practice, more than half of the participants (52.68%) reported that their children were relatively compliant with the physician’s advice but not fully compliant (P1). In terms of paying attention to their child’s emotions (P3), giving proactive advice to improve emotions (P4), and encouraging their child to participate in social activities (P5), 36.40, 27.01, and 38.12% of parents, respectively, demonstrated they were well aligned with those practices. Notably, 51.35% stated that they were likely to argue with their children (P6), and 44.64% reported that they were likely to refuse their child’s requests (P7). In addition, 53.06% of the participants showed a trend of taking the initiative to learn about adolescent depression (P8). The most frequently cited sources of information were the Internet (77.39%) and healthcare providers (56.51%) (P9) (Supplementary Table S4).

Significant positive correlations were found between knowledge and attitude (*r* = 0.225, *p* < 0.001), knowledge and practice (*r* = 0.240, *p* < 0.001), and attitude and practice (*r* = 0.381, *p* < 0.001) (Table 2).

**Table 2:** Correlation analysis.

	Knowledge	Attitude	Practice
Knowledge	1		
Attitude	0.225 (*p* < 0.001)	1	
Practice	0.240 (*p* < 0.001)	0.381 (*p* < 0.001)	1

The pathway (Table 3) and direct/indirect effects (Table 4) analyses showed that there were direct effects of knowledge on attitude (β = 0.422, *p* < 0.001), and practice (β = 0.283, *p* < 0.001) and also an indirect effect through attitude (β = 0.131, *p* = 0.004) on practice. At the same time, the attitude was also directly affecting practice (β = 0.311, *p* < 0.001).

**Table 3:** Path coefficients.

	Estimate	S.E.	C.R.	*p*-value
Knowledge→Attitude	0.422	0.074	5.730	<0.001
Knowledge→Practice	0.283	0.067	4.237	<0.001
Attitude→Practice	0.311	0.038	8.079	<0.001

**Table 4:** Direct effects and indirect effects.

	Total effects		Direct effects		Indirect effects	
	Effect	*p*-value	Effect	*p*-value	Effect	*p*-value
Knowledge→Attitude	0.422		0.422	<0.001		
Knowledge→Practice	0.414	0.004	0.283	<0.001	0.131	0.004
Attitude→Practice	0.311		0.311	<0.001		

## Discussion

Parents of adolescents diagnosed with depression exhibited moderate knowledge, attitudes, and practices regarding adolescent depression. Hence, targeted interventions should be recommended to enhance parental awareness, reduce stigma, and improve communication about adolescent depression. Emphasis should be placed on imparting practical skills for effective support, including recognizing warning signs and facilitating access to mental health resources. Promoting collaboration among healthcare providers, mental health professionals, educators, and parents might be crucial for adopting a holistic approach. By working together, these stakeholders could contribute to a comprehensive support system that addresses the adolescents’ immediate needs and fosters a supportive environment for long-term mental health and well-being.

The study highlights that parents of adolescents diagnosed with depression demonstrated a moderate level of knowledge, attitudes, and practices concerning the condition. Notably, variations in knowledge, attitude, and practice scores were observed among parents based on residence, education levels, gender, occupation, monthly *per capita* income, number of children, and the severity of their child’s depression. These findings could help identify parents with poorer KAP who require targeted educational intervention to improve their KAP. As reported in a previous study, parental change led to changes in children’s self-understanding and depressive symptomatology (15). Therefore, the observed variations across demographic factors reinforce the need for nuanced strategies that consider the unique challenges and perspectives of different parent groups, ultimately contributing to more effective clinical practices in supporting adolescents with depression.

The results indicated a variable level of knowledge among parents regarding adolescent depression. While the present study reported a high correct rate for recognizing the impact of depression on work, education, and social functioning and the importance of early diagnosis and timely treatment, it is crucial to address certain misconceptions that are evident. These findings underscored the need for targeted interventions to correct these misconceptions and ensure a comprehensive understanding of adolescent depression among parents. Notably, a considerable proportion believed that all cases of depression require hospitalization, and they showed misconceptions about the addictiveness of antidepressant medications. Targeted educational interventions are essential to address these deficiencies. Emphasis should be placed on dispelling common myths surrounding depression treatment. A previous study emphasized the efficacy of educational interventions in correcting misconceptions and improving knowledge about mental health issues (16). Integrating such interventions into routine healthcare practices, perhaps through informational sessions or pamphlets, could contribute to a more accurate understanding of adolescent depression among parents and, consequently, lead to improved clinical outcomes (17, 18).

The attitude assessment revealed diverse perspectives among parents of adolescents diagnosed with depression. While a significant proportion expressed concern and a desire to understand the reasons behind their child’s depression, some attitudes suggested potential challenges in the parent–child dynamic. Notably, a considerable number felt bothered and ashamed or questioned their competence as parents in response to their child’s depression. In order to address these complex attitudes, interventions should focus on fostering empathy, reducing stigma, and enhancing parental coping strategies. Educational programs could be designed to provide information on the nature of depression, its causes, and the importance of supportive parenting. Emphasizing the role of open communication in understanding and addressing parental concerns may contribute to a more constructive attitude (19, 20). Previous studies showed the efficacy of interventions aimed at reducing stigma and improving attitudes toward mental health issues (21, 22). Incorporating these elements into clinical practice could help create a more supportive environment for both parents and adolescents coping with depression.

The examination of parental practices in the context of adolescent depression revealed a mixed spectrum of behaviors. While many parents followed treatment recommendations, paid active attention to their child’s emotions, and encouraged social engagement, areas of concern are evident. Notably, many parents reported frequently arguing with their children and denying their children’s requests, indicating potential challenges in parent–child interactions. Interventions should focus on enhancing family communication and conflict resolution skills to address these problems in practice. Parenting programs that emphasize positive communication strategies and stress the importance of maintaining a supportive environment for adolescents with depression may prove beneficial (23, 24). Previous studies highlighted the effectiveness of such interventions in improving family dynamics and fostering positive outcomes for adolescents with mental health challenges (25, 26). In addition, efforts should be directed toward increasing awareness about reliable sources of information on adolescent depression (27, 28).

The correlation and pathway analyses revealed intricate relationships among parental knowledge, attitudes, and practices concerning adolescent depression. Positive correlations were identified between knowledge and attitude, between knowledge and practice, and between attitude and practice, indicating that higher levels of knowledge were associated with more positive attitudes and practices. The pathway analysis further elucidated these associations, demonstrating the direct effects of knowledge on attitude and practice. Furthermore, attitude exhibited a direct impact on practice, and there was an indirect effect of knowledge on practice through attitude. Hence, interventions should adopt a comprehensive approach targeting not only knowledge enhancement but also the cultivation of positive attitudes (29, 30). Educational programs focusing on factual information about depression and strategies to foster supportive attitudes may yield favorable outcomes. A previous study supports that interventions combining knowledge enhancement and attitude change could be particularly successful in influencing health-related behaviors (31). Implementing such multifaceted interventions in clinical practice can contribute to a more holistic and impactful approach to supporting adolescents with depression and their families.

This study had several limitations. Firstly, the questionnaire was tested for construct validity after the formal survey. Secondly, the study was conducted in a single center, thus limiting its generalizability. Thirdly, the questionnaire did not examine whether the parents had more than one child diagnosed with depression. Lastly, the data were collected via self-administered questionnaires, which may introduce biases such as self-report and social desirability bias.

In conclusion, parents of adolescents diagnosed with depression showed a moderate KAP toward adolescent depression. Targeted interventions could be recommended to improve parental awareness, reduce stigma, and enhance communication regarding adolescent depression. Educational programs should emphasize practical skills for effective support, including recognizing warning signs and facilitating access to mental health resources. While the findings underscored the importance of these interventions, further research is needed to explore the potential collaborative efforts among healthcare providers, mental health professionals, educators, and parents in addressing the complex relationship between parental KAP and adolescent depression.

## Data availability statement

The original contributions presented in the study are included in the article/Supplementary material, further inquiries can be directed to the corresponding author.

## Ethics statement

The studies involving humans were approved by Ningbo First Hospital Ethics Committee (No. 2022-016A). The studies were conducted in accordance with the local legislation and institutional requirements. The participants provided their written informed consent to participate in this study.

## Author contributions

HG: Writing – review & editing, Writing – original draft, Methodology, Investigation, Formal analysis, Data curation. ND: Writing – review & editing, Investigation. CL: Writing – review & editing, Investigation. YY: Writing – review & editing, Formal analysis, Data curation. DY: Writing – review & editing, Formal analysis, Data curation. QZ: Writing – review & editing, Investigation, Data curation. JM: Writing – review & editing, Investigation, Data curation. ZL: Writing – review & editing, Project administration. YJ: Writing – review & editing, Project administration. LR: Writing – review & editing. YH: Writing – review & editing, Validation, Conceptualization.
