# Supplementary material for: Knowledge, attitudes, and practices of adolescent depression among parents of children diagnosed with depression in Ningbo City, eastern China
Source: Front Public Health. 2024 Jun 11;12:1404819. doi: 10.3389/fpubh.2024.1404819 (PMC11196748; doi:10.3389/fpubh.2024.1404819)
Supplement: Supplementary file 1 [file Data_Sheet_1.doc]

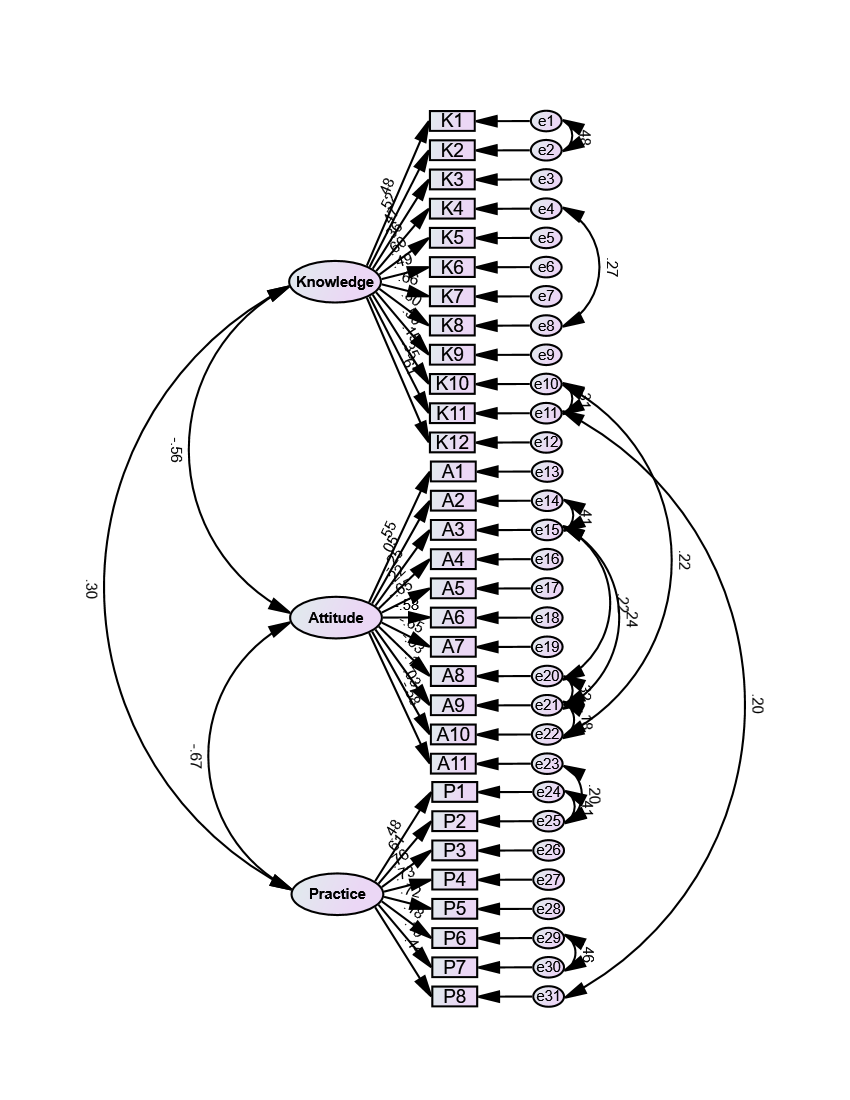


**Supplementary Figure 1. Confirmatory factor analysis**

**Supplementary Table 1. Model fit indicators for confirmatory factor analysis**

| Indicators | Reference | Actual |
| --- | --- | --- |
| CMIN/DF | 1-3: Excellent, 3-5: Good | 2.640 |
| RMSEA | <0.08: Good | 0.056 |
| IFI | >0.8: Good | 0.833 |
| TLI | >0.8: Good | 0.812 |
| CFI | >0.8: Good | 0.831 |

**Supplementary Table 2. Knowledge**

|  | **Correct Rate** **N (%)** |
| --- | --- |
| **1.** **Depression is a common mental disorder, and long-term moderate to severe depression can significantly impact a person's work, education, and social functioning, potentially leading to suicidal thoughts or actions.** | 454 (86.97) |
| **2.** **Symptoms of adolescent depression may include self-blame, low self-confidence, reduced self-esteem, insomnia, binge eating or loss of appetite, self-harming behaviors, suicidal thoughts, aggression, emotional sensitivity, and more.** | 472 (90.42) |
| **3.** **Believing that childhood depression is just an emotional issue and does not require treatment is incorrect.** | 468 (89.66) |
| **4.** **Having many friends does not necessarily protect a child from developing depression.** | 274 (52.49) |
| **5.** **Issues within the family system, such as family relationships, parenting styles, and family functioning, can contribute to the progression of adolescent depression.** | 446 (85.44) |
| **6.** **Signs like suicidal statements, suicide attempts, worsening depression or anxiety, restlessness, panic attacks, insomnia, irritability, impulsiveness, and manic behavior suggest a worsening of depression and the need for prompt medical attention.** | 491 (94.06) |
| **7.** **Early diagnosis and timely treatment of depression can improve treatment outcomes.** | 496 (95.02) |
| **8.** **All cases of depression require hospitalization.** | 294 (56.32) |
| **9.** **Psychological counseling is an effective form of cognitive-behavioral therapy for treating depression.** | 437 (83.72) |
| **10.** **Antidepressant medications are addictive.** | 151 (28.93) |
| **11.** **It is essential to follow a medical professional's guidance when discontinuing antidepressant medication, even if one's mood improves.** | 274 (52.49) |
| **12.** **Behaviors such as maintaining a regular diet, engaging in physical activity, getting sufficient sleep, developing interests, and communicating with others can have a positive impact on improving depression.** | 490 (93.87) |

**Supplementary Table 3. Attitude**

|  | **N (%)** | | | | |
| --- | --- | --- | --- | --- | --- |
|  | **Strongly Agree** | **Agree** | **Neutral** | **Disagree** | **Strongly Disagree** |
| **1.** **Your child having depression causes you a great deal of concern.** | 351 (67.24) | 141 (27.01) | 24 (4.60) | 2 (0.38) | 4 (0.77) |
| **2.** **Your child having depression makes you feel bothered.** | 123 (23.56) | 126 (24.14) | 86 (16.48) | 129 (24.71) | 58 (11.11) |
| **3.** **Your child having depression makes you feel ashamed.** | 22 (4.21) | 29 (5.56) | 98 (18.77) | 252 (48.28) | 121 (23.18) |
| **4.** **Your child's depressive episodes make you question your competence as a parent.** | 97 (18.58) | 232 (44.44) | 93 (17.82) | 90 (17.24) | 10 (1.92) |
| **5.** **You want to understand the reasons behind your child's depression.** | 278 (53.26) | 207 (39.66) | 28 (5.36) | 9 (1.72) | / |
| **6.** **You believe that pressuring your child to do things when they are feeling down or depressed would worsen their psychological state.** | 214 (41.00) | 246 (47.13) | 25 (4.79) | 29 (5.56) | 8 (1.53) |
| **7.** **You believe that pressuring your child to do things when they are feeling down or depressed would worsen their psychological state.** | 305 (58.43) | 199 (38.12) | 13 (2.49) | 3 (0.57) | 2 (0.38) |
| **8.** **You view a child's depression as a sign of emotional weakness.** | 32 (6.13) | 139 (26.63) | 77 (14.75) | 235 (45.02) | 39 (7.47) |
| **9.** **You wish for your child to strictly follow your requirements.** | 15 (2.87) | 55 (10.54) | 169 (32.38) | 242 (46.36) | 41 (7.85) |
| **10.** **You worry that the side effects of antidepressant medication may affect your child's development.** | 77 (14.75) | 220 (42.15) | 141 (27.01) | 78 (14.94) | 6 (1.15) |
| **11.** **You have trust in the treatment recommendations provided by the doctor for your child.** | 214 (41.00) | 287 (54.98) | 20 (3.83) | 1 (0.19) | / |

**Supplementary Table 4. Practice**

|  | **N (%)** | | | | |
| --- | --- | --- | --- | --- | --- |
|  | **Well aligned** | **Aligned** | **Neutral** | **Not aligned** | **Not aligned at all** |
| **1.** **Your child strictly follows the doctor's advice on taking medication or receiving psychological counseling.** | 183 (35.06) | 275 (52.68) | 55 (10.54) | 6 (1.15) | 3 (0.57) |
| **2.** **You faithfully adhere to the doctor's recommendations and regularly take your child for follow-up appointments.** | 203 (38.89) | 269 (51.53) | 38 (7.28) | 11 (2.11) | 1 (0.19) |
| **3.** **You actively pay attention to your child's emotions and inquire about their feelings.** | 190 (36.40) | 274 (52.49) | 46 (8.81) | 9 (1.72) | 3 (0.57) |
| **4.** **You voluntarily offer your child suggestions for improving their mood.** | 141 (27.01) | 290 (55.56) | 70 (13.41) | 17 (3.26) | 4 (0.77) |
| **5.** **You encourage your child to participate in social activities.** | 199 (38.12) | 277 (53.07) | 33 (6.32) | 10 (1.92) | 3 (0.57) |
| **6.** **You frequently argue with your child.** | 20 (3.83) | 51 (9.77) | 197 (37.74) | 194 (37.16) | 60 (11.49) |
| **7.** **You often deny your child's requests.** | 11 (2.11) | 26 (4.98) | 196 (37.55) | 242 (46.36) | 47 (9.00) |
| **8.** **You regularly seek out information on adolescent depression.** | 97 (18.58) | 180 (34.48) | 170 (32.57) | 60 (11.49) | 15 (2.87) |
| **9.** **You are aware of the ways to obtain information about depression. (Select all that apply)** |  |  |  |  |  |
| **a.** **Relatives and friends** | 166 (31.80) |  |  |  |  |
| **b.** **Healthcare professionals** | 295 (56.51) |  |  |  |  |
| **c. Internet (websites, mobile apps like WeChat, etc.)** | 404 (77.39) |  |  |  |  |
| **d.** **Television** | 112 (21.46) |  |  |  |  |
| **e.** **Radio** | 59 (11.30) |  |  |  |  |
| **f.** **Magazines and newspapers** | 89 (17.05) |  |  |  |  |
| **g.** **I have never actively sought information** | 19 (3.64) |  |  |  |  |
| **h. Other** | 18 (3.45) |  |  |  |  |
